# An immunogenic cell death-related lncRNA signature correlates with prognosis and tumor immune microenvironment in bladder cancer

**DOI:** 10.1038/s41598-024-63852-9

**Published:** 2024-06-07

**Authors:** Jinhong Luo, Feiye Luo, Qin Li, Qinghong Liu, Jinshan Wang

**Affiliations:** 1 Department of Oncology, East Hospital, Tongji University School of Medicine, No. 1800 Yuntai Road, Shanghai, 200123 China; 2 Department of Oncology, East Hospital, Ji’an Hospital, Ji’an, 343000 Jiangxi China; 3 Department of Urology, Dongfang People’s Hospital, Dongfang, 572699 Hainan Province China

**Keywords:** Bladder cancer, Immunogenic cell death, lncRNAs, Nomogram, TCGA, Tumor Immune Microenvironment, Cancer, Cell biology

## Abstract

Immunogenic cell death (ICD) is a newly discovered form of cellular demise that triggers adaptive immune responses mediated by T cells. However, the immunogenic cell death-related lncRNAs (ICDRLs) involved in bladder cancer (BC) development and progression remain to be further elucidated. Molecular profiling data and clinicopathological information for BC patients were obtained from TCGA, and the ICDRGs list was obtained from published literature. For the identification of ICDRLs, Pearson co-expression analysis was performed, and a prognostic signature based on 13 ICDRLs was constructed by univariate assays and LASSO assays. Herein, an ICDRLSig consisting of 13 ICDRLs was constructed. KM curves and ROC curves demonstrated that the constructed signature in the TCGA training, testing, entire and external sets have good predictive performance. Multivariate assays illuminated that the signature is an independent predictor for BC patients’ OS, exhibiting greater predictive power for the survival than traditional clinicopathological features. Additionally, patients in the high-ICDRLSig risk subgroup had more abundant immune infiltration, higher immune checkpoint gene expression, lower TMB and poorer response to immunotherapy. We have developed a novel ICDRLSig that can be exploited for survival prediction and provide a reference for further individualized treatment.

## Introduction

Bladder cancer (BC) is one of the deadly urological malignancies^1,2^. Local excision and intravesical chemotherapy or intravesical Bacillus Calmette-Guerin (BCG) immunotherapy are the most frequent treatment strategy for patients with NMIBC^3,4^. Although NMIBC patients have a relatively good prognosis compared with MIBC patients, some patients still experience recurrence or even progression to MIBC, requiring long-term monitoring and repeated surgical intervention^5^. MIBC requires radical surgery, including resection of the bladder and bilateral pelvic lymph nodes, but related complications, such as intestinal obstruction and anastomotic leakage, are still significant problems^6,7^. Although the clinical treatments of BC have improved over the past decade, the prognosis of BC patients is still disappointing^8,9^. Furthermore, the lack of recognized prognostic biomarkers for BC makes it difficult for urologists to perform risk stratification and prognostic assessment of patients. Over the past decade, gene signatures have been widely used for risk stratification and prognostic research of cancer patients. An effective prognostic signature is urgently needed to predict the survival outcome of patients with BC.

Immunogenic cell death (ICD) is regulated cell death (RCD) induced by certain chemotherapy, radiation therapy, oncolytic viruses, physical chemotherapy, and photodynamic therapy^10^. Research has shown that after induction of immunogenic cell death, tumor antigens and damage-associated molecular patterns (DAMPs) released by dying tumor cells, which activate pattern recognition receptors (PRRs) on dendritic cells (DCs), thereby activating T cells to produce anti-tumor immune responses^11,12^. immunogenic cell death can kill not only cells induced by immunogenic cell death inducers, but also dying cancer cells as tumor vaccines, thereby enhancing the therapeutic effect of conventional anticancer chemotherapy and radiotherapy^13^. So far, several immunogenic cell death inducers have been successfully used in BC preclinical studies. For example, N-vanillyl-8-methyl-nonenamide (CPS) can induce immunogenic cell death in human BC cells^14^. Norcantharidin (NCTD) can induce immunogenic cell death (ICD), thereby promoting anticancer immune responses^15^. However, the existing evidence for their clinical utility is not convincing. Therefore, further studies in patients should be performed in a clinical setting to assess the possibility of immunogenic cell death.

The whole sequencing technology helps to find out that 98% of mammalian genome are located in the non-coding RNAs of protein, and only 2% in the encoded region^16^. By definition, LncRNAs are non-coding RNA molecules with over 200 nucleotides^17^. Long non-coding RNAs (lncRNAs) were able to regulate gene transcription and post-transcription, leading to cancer initiation and progression^18^. In BC, lncRNAs serve various crucial roles in the proliferation, invasion and prognosis of tumor cells^18–20^. In clinical studies, it has been reported that many lncRNAs can be potential biomarkers to predict the diagnosis and prognosis of cancer in different tumors, including BC^21,22^. However, no study has addressed the role of ICDRLs in BC.

In this study, we aimed to developed a novel prognostic model based on ICDRLs. Here, we systematically assessed the ICDRLs identified in BC. A prognostic signature based on 13 ICDRLs was constructed for BC patients, and its correlation with immune and mutation landscape, chemotherapy and immunotherapy was investigated. In addition, we sought to develop an improved way of predicting BC patients' prognosis as well as the effect of their treatment.

## Materials and methods

### Data collection

The transcriptomic data, somatic mutation data, and clinicopathological information of BC was downloaded from TCGA datasets. Only 396 patients with a survival of more than 30 days were encompassed in our research. Additionally, the immunogenic cell death-related genes (ICDRGs) were obtained from a previous literature (Table S1)^23^. Furthermore, the dataset GSE13507 was acquired from the Gene Expression Omnibus (GEO) database (https://www.ncbi.nlm.nih.gov/geo/) and utilized as a validation set. It was derived from the Illumina Human-6 v2.0 Expression BeadChip platform^24^.

### Model building, evaluation and clinical significances

Preliminarily, Pearson’s test was carried out to explore the correlation between ICDRGs and immunogenic cell death-related lncRNAs (ICDRLs) (|R|> 0.4 and *p* < 0.001). ICDRLs associated with overall survival were identified using univariate assays with the “survival” R package (*p* < 0.01). Further, we randomly grouped BC patients into a training and validation set at a ratio of 7:3. An immunogenic cell death-related lncRNA signature (ICDRLSig) was constructed using LASSO Cox regression analysis in TCGA training cohort using the “glmnet” package. Lasso regression demonstrated that cross-validation was best when λ = − 3.9, and 13 lncRNAs were included for the construction of prognostic models. Then, the risk score for each and every patient diagnosed with BC was determined using the computational formula: risk score = (normalized expression level of each ICDRL × corresponding regression coefficient). Patients in the training set, testing set, and the whole set and external validation set were then classified into high- and low- ICDRLSig risk subgroups, using median score of the training set as cutoffs. KM curves were used to estimate survival outcomes between differed risk subgroups and time-dependent ROC curves were generated to assess the predictability and reliability of the established ICDRLSig. Univariate and multivariate Cox proportional hazards regression model was applied to evaluate the independence of the ICDRLSig. It was determined that the accuracy of a prediction nomogram that was developed on the basis of independent prognostic criteria could be improved through the use of calibration curves. Calculating the areas under the ROC curves allowed for a comparison of the diagnostic efficacy of risk scores and clinicopathological variables (AUCs). In addition to this, we investigated the differences in risk ratings between clinical characteristics.

### Functional enrichment analysis of the ICDRLSig

The “limma” package was used to screen the differentially expressed genes across the high- and low-ICDRLSig risk subgroups (false discovery rate (FDR) 0.05 and |log2 fold change (FC)|> 1), and the “clusterProfiler” R package was used to functionally annotate the genes based on the GO and KEGG^25^. In addition, cluster analysis was carried out using the “clusterProfiler” R program (NOM P was less than 0.05, and FDR was also less than 0.05).

### The immune and mutation landscapes of the ICDRLSig

Immune cell scores, immune pathway scores, and TME scores are calculated via the “GSVA” R package^26^ and “ESTIMATE” algorithm^27^, respectively. We analyzed the differences in immune cell scores, immune pathway scores, and TME scores between high-risk persons and low-risk individuals in order to investigate if there are variations in immunological state between high-risk and low-risk groupings. As a defense mechanism, tumor cells express immunological checkpoint molecules. Therefore, we compared the high-risk/low-risk subgroups’ expression levels of immunological check inhibitory substances. To determine whether low-risk and high-risk populations exhibit different somatic mutations. The “maftools” R package^28^ was employed to visualize mutation data and calculate TMB for patients with BC in TCGA.

### Analysis of drug sensitivity and immunotherapy response

The “pRRophetic” R package^29^ was used to predict the half-maximal inhibitory concentrations (IC50) of common drugs and to assess the sensitivity of chemotherapy and targeted therapy between the two risk subgroups. Additionally, to assess the immunotherapy sensitivity in patients with different risk groups, the immunophenoscore (IPS) scores were downloaded from TCIA for predicting immune checkpoint blockade (ICB) responses in different risk subgroups (anti-PD-1 and anti-CTLA4)^30,31^.

### Statistical analysis

All statistical analyzation involved were completed using R software (Version 4.1.2). A value of *p* < 0.05 was regarded as significantly different.

## Results

### Identification of prognostic ICDRLs in BC

**Figure 1:**
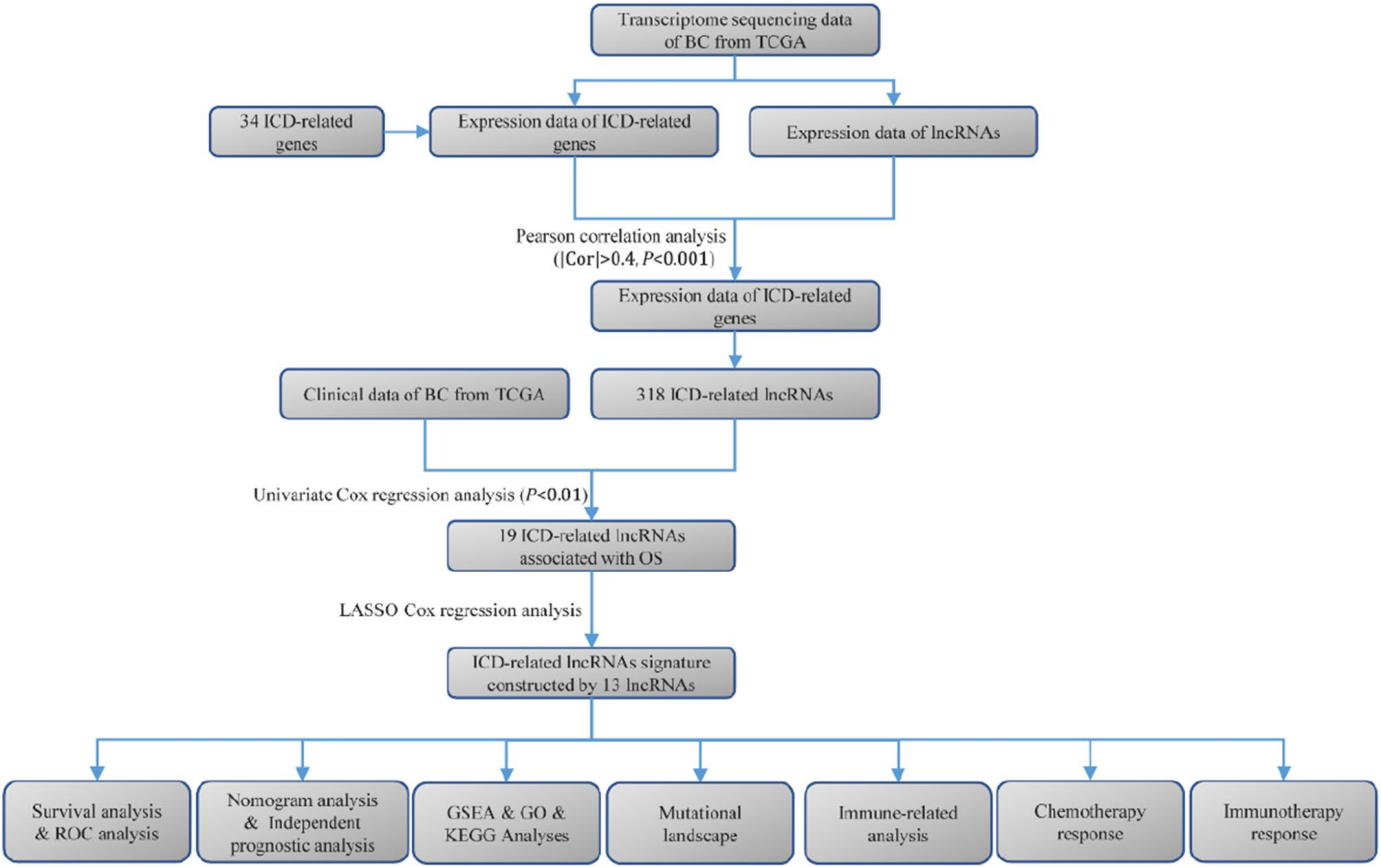
Flow chart of present study.

**Figure 2:**
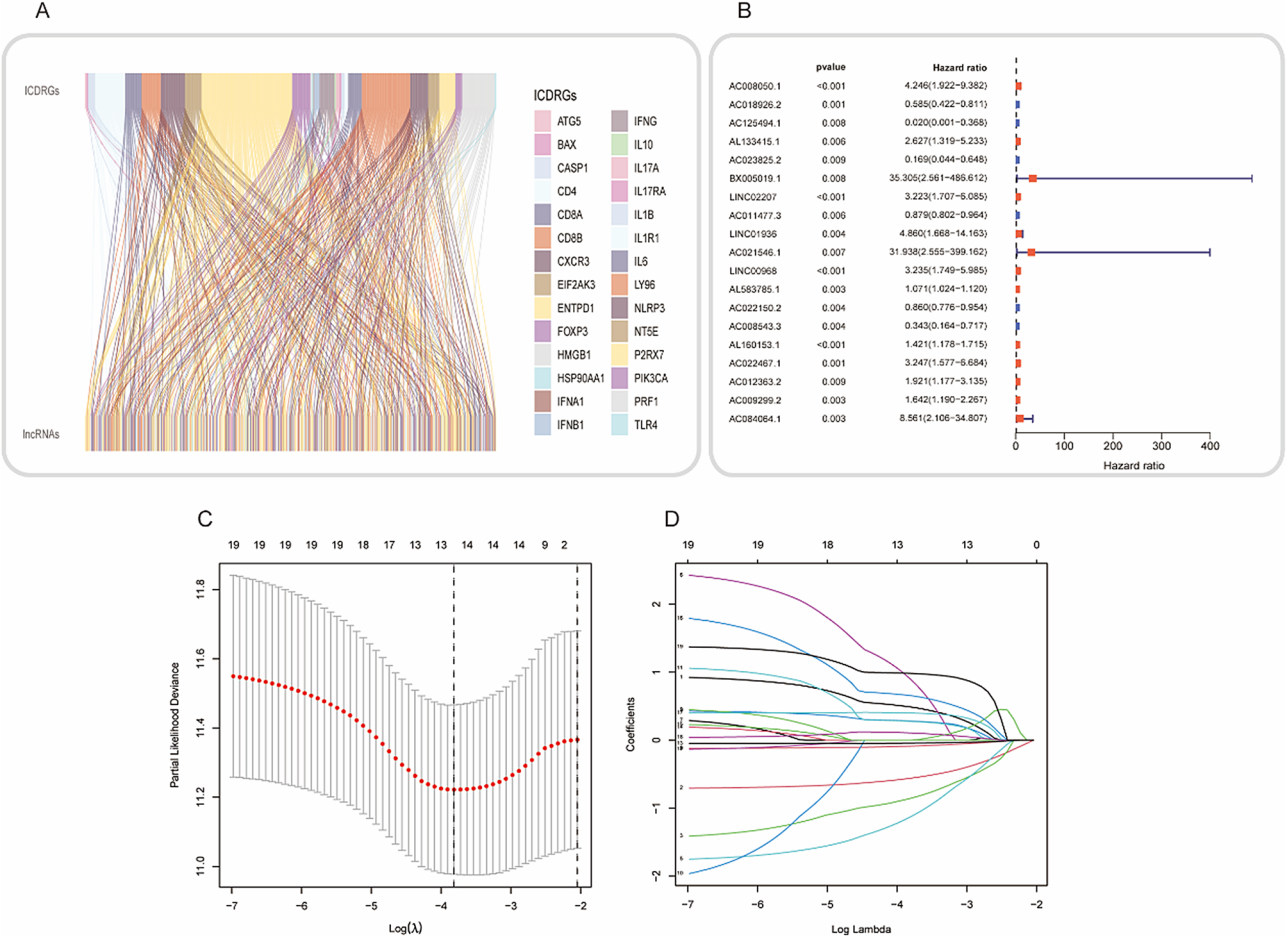
Screening for model development of prognostic immunogenic cell death-related lncRNAs. (**A**) A Sankey diagram depicting the network of lncRNAs and genes relevant to immunogenic cell death. (**B**) A univariate Cox analysis was performed, and the results showed that immunogenic cell death-related lncRNAs were correlated with overall survival. (**C**) LASSO Cox regression. (**D**) Determine the best parameters for the LASSO filter.

Figure 1 illustrated the overall workflow of this study. Herein, 34 ICDRGs (HMGB1, HSP90AA1, ATG5, ENTPD1, NT5E, CALR, BAX, CASP8, PDIA3, EIF2AK3, IFNA1, IFNB1, PIK3CA, CXCR3, IL10, IL6, TNF, CASP1, IL1R1, IL1B, NLRP3, IFNG, IFNGR1, P2RX7, LY96, MYD88, TLR4, FOXP3, IL17A, IL17RA, PRF1, CD4, CD8A, and CD8B) were selected for further analysis. We then selected the 318 ICDRLs according to the cutoff criteria of Pearson |R|> 0.4 and *p* < 0.001. Furthermore, an mRNA-lncRNA network was constructed based on the significant correlation pairs (Fig. 2A). Univariate Cox proportional hazards regression was performed on 318 ICDRLs and 19 prognostic ICDRLs in BC were ultimately determined (Fig. 2B and Table S2). Lasso regression was applied to establish a prediction model based on 13 ICDRLs (AC008050.1, AC018926.2, AC125494.1, AL133415.1, AC023825.2, BX005019.1, AC011477.3, LINC00968, AC022150.2, AC022467.1, AC012363.2, AC009299.2, and AC084064.1) in the training set (Fig. 2C,D). Based on the expressions of the 13 ICDRLs and the corresponding weighted coefficients, the risk score of BC patients were calculated based pm the following formula: risk score = (0.4895 × AC008050.1) + (− 0.5616 × AC018926.2) + (− 0.8580 × AC125494.1) + (0.2821 × AL133415.1) + (− 1.1317 × AC023825.2) + (0.9036 × BX005019.1) + (− 0.0881 × AC011477.3) + (0.2766 × LINC00968) + (− 0.0441 × AC022150.2) + (0.6564 × AC022467.1) + (0.3992 × AC012363.2) + (0.1064 × AC009299.2) + (0.9827 × AC084064.1).

### Assessment and validation of the ICDRLSig

**Figure 3:**
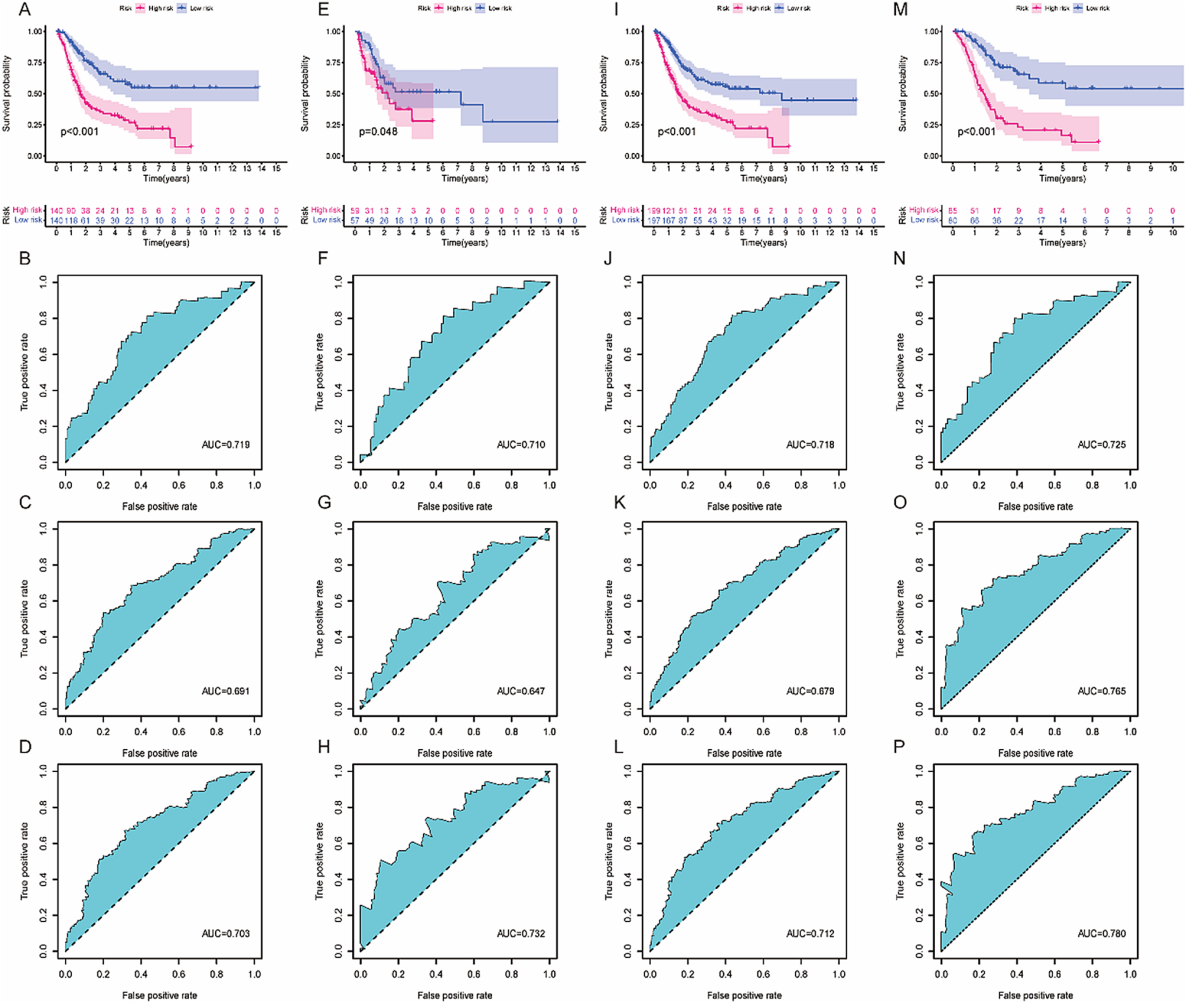
Evaluation and confirmation of the predictive value of the lncRNA signature associated with immunogenic cell death. (**A**, **E**, **I**, **M**) The Kaplan–Meier survival curves of the low-risk group, on the one hand, and the high-risk group, on the other, for the TCGA training set, the testing set, and the full set, as well as the external validation set, respectively. (**B**, **C**, **D**) ROC curves were used to evaluate how well the risk signature could predict survival rates of 1 year, 3 years, and 5 years in the TCGA training set. (**F**, **G**, **H**) ROC curves were used to evaluate how well the risk signature could predict survival rates at 1 year, 3 years, and 5 years in the TCGA testing set. (**J**, **K**, **L**) ROC curves were used to evaluate how well the risk signature could predict survival rates at 1 year, 3 years, and 5 years in the TCGA complete cohort. (**N**, **O**, **P**) ROC curves were used to evaluate how well the risk signature could predict survival rates at 1 year, 3 years, and 5 years in the external validation set.

The method that had been presented before was used to calculate each patient's risk score, which was then presented. After that, we used the median risk score from the training set as a cut-off value to dichotomize the training set, as well as the testing set and the whole set, and the external validation set, into low-risk and high-risk subsets. This was done using the training set. According to the Kaplan–Meier survival curves, patients in the high-risk subgroup had a shorter overall survival time compared to patients (Fig. 3A,E,I,M). ROC curves were developed in order to evaluate the performance of the established signature in terms of its ability to predict the OS of patients with BC. In the training cohort, the areas under the curve (AUCs) for 1 year, 3 years, and 5 years, respectively, were 0.719, 0.691, and 0.707, respectively (Fig. 3B,C,D). Within the population represented by the sample, the areas under the curve (AUCs) for 1 year, 3 years, and 5 years were respectively 0.710, 0.647, and 0.732. (Fig. 3F,G,H). The areas under the curve (AUCs) for the overall cohort were 0.718, 0.679, and 0.712, respectively, for 1 year, 3 years, and 5 years of follow-up, respectively (Fig. 3J,K,L). The areas under the curve (AUCs) for the external cohort were 0.725, 0.765, and 0.780, respectively, for 1 year, 3 years, and 5 years of follow-up, respectively (Fig. 3N,O,P).

### Clinical implications of the ICDRLSig

**Figure 4:**
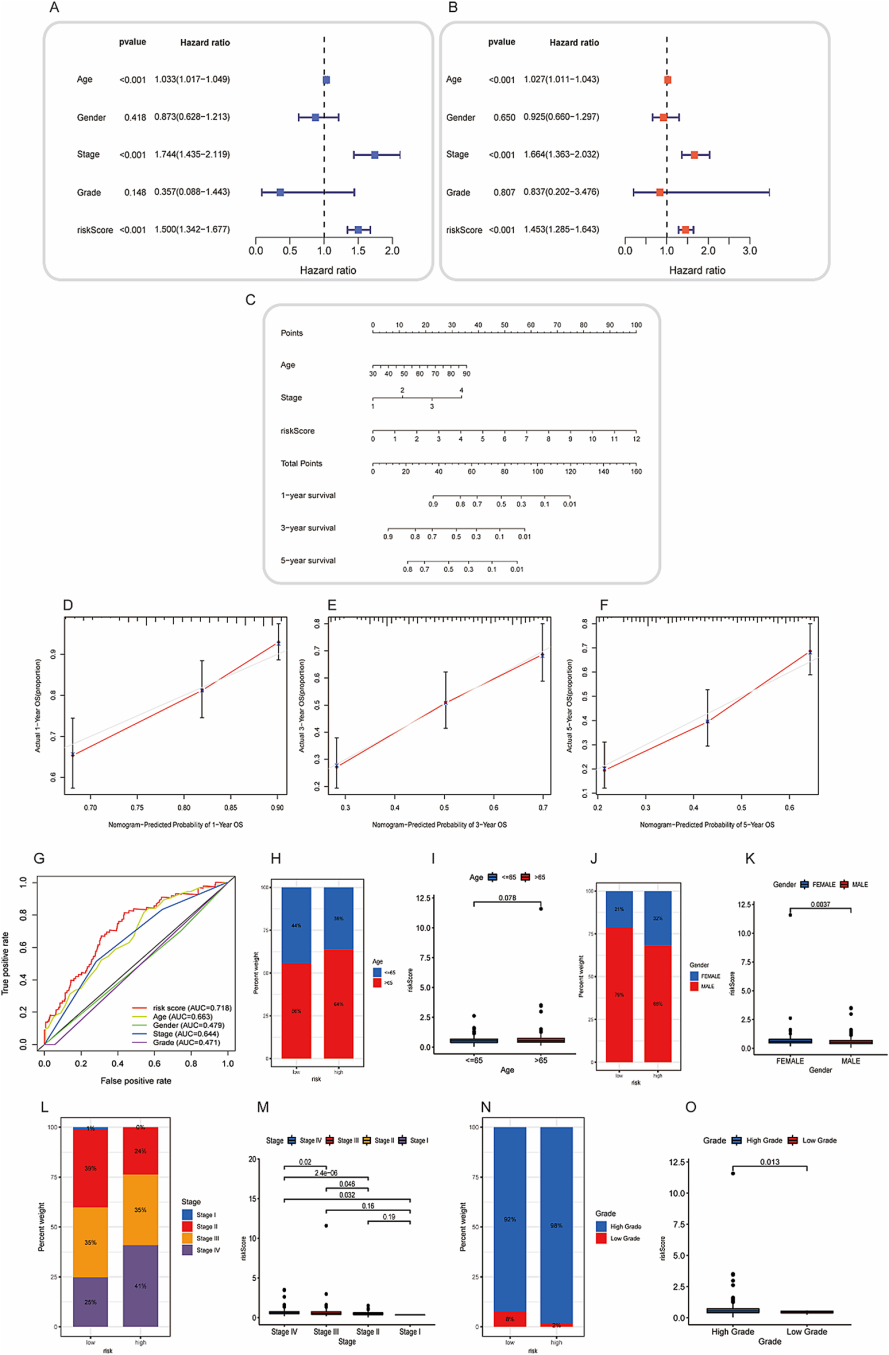
Prognostic value of the immunogenic cell death-related lncRNA signature (ICDRLSig) (**A**, **B**) Univariate and multivariate assays of the established signature as an independent prognostic factor using the Cox regression method. (**C**) A predictive nomogram that takes into account various clinicopathological parameters in addition to the ICDRLSig. (**D**–**F**) Survival rate calibration curves of the line chart for 1 year, 3 years, and 5 years respectively. (**G**) ROC curves that take into account both the clinical features and the risk score. (**H**–**O**) The relationship between the risk scores and the clinicopathological variables.

In this research, we analyze the clinical relevance of the ICDRLSig by completing numerous analyses, which are mentioned throughout the article. To begin, univariate and multivariate assays were done with the purpose of evaluating the degree to which the ICDRLSig risk score is independent from other variables in determining prognosis. According to the results of a univariate Cox regression analysis, the risk score was considerably related with OS in patients with BC (Fig. 4A). In BC patients, a multivariate Cox regression analysis demonstrated that the risk score maintained to be an independent predictor of overall survival (OS), even after taking into account a variety of clinical characteristics (Fig. 4B). Among other criteria, age and stage were also revealed to be independent predictive variables for OS of BC patients. In the second step of the process, a nomogram was built to examine the clinical value of the ICDRLSig. This nomogram was able to predict overall survival at 1, 3, and 5 years (Fig. 4C). The calibration plots indicated that there was a good concordance between the survival probability that was predicted by the nomogram and the observations that were made (Fig. 4D,E,F). Furthermore, the ROC curve analysis suggested that the AUC value of the ICDRLSig was 0.718, which was higher than the AUC values of other clinical factors (Fig. 4G). Finally, the relationships between the ICDRLSig risk score with various clinical features of BC patients were also analyzed, and we found that the ICDRLSig risk score in elderly patients (> 65 years old), female, Stage IV, and high grade was higher than those less than 65 years old, male, Stage I-III, and low grade (Fig. 4H–O). There was no correlation at all seen between age and the ICDRLSig risk score.

### Functional signaling exploration of the ICDRLSig

**Figure 5:**
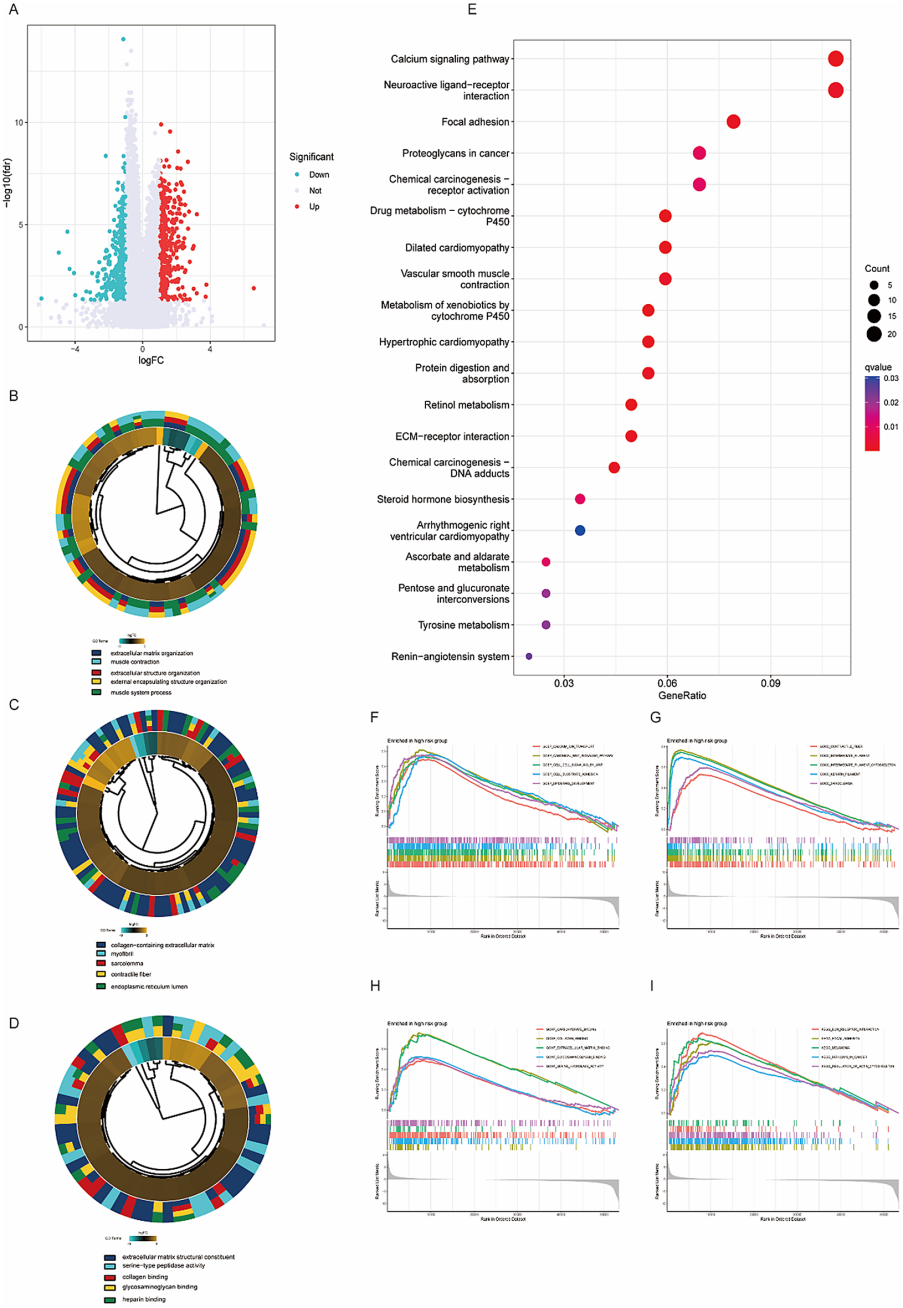
Functional analysis of the immunogenic cell death-related lncRNA signature (ICDRLSig). Genes that are expressed in a way that is distinct between high-risk and low-risk populations (**A**). The results shown here are representative of the GO (**B**–**D**) and KEGG (**E**) analyses performed on the ICDRLSig. GSEA of high-risk group based on ICDRLSig (**F**–**I**).

To investigate the potential functional roles of the ICDRLSig, 942 differentially expressed DEGs between the two risk subgroups were analyzed by functional enrichment analysis (Fig. 5A). As shown in Fig. 5B,C,D, the top five enriched GO terms in biological processes were extracellular matrix organization, extracellular structure organization, muscle system process, and muscle contraction, and those in cellular components were collagen-containing extracellular matrix, sarcolemma, contractile fiber, and myofibril, regarding molecular function, DEGs were significantly enriched in terms of extracellular matrix structural constituent, collagen binding, collagen binding, heparin binding, and serine-type peptidase activity. KEGG pathway analysis of DEGs revealed enrichment for calcium signaling pathway, neuroactive ligand-receptor interaction, focal adhesion, proteoglycans in cancer, and Chemical carcinogenesis-receptor activation (Fig. 5E). Additionally, GSEA was further performed to illustrate the ICDRLSig regulation of BC progression. The results revealed that the top five BP terms substantially enriched in the high-ICDRLSig risk subgroup were calcium ion transport, canonical Wnt signaling pathway, cell signaling by Wnt, cell-substrate adhesion, and epidermis development (Fig. 5F), the results of CC indicated that contractile fiber, intermediate filament, intermediate filament cytoskeleton, keratin filament, and sarcolemma (Fig. 5G), as for MF, the high-ICDRLSig risk subgroup was enriched in carbohydrate binding, collagen binding, extracellular matrix binding, glycosaminoglycan binding, and serine hydrolase activity (Fig. 5H). Additionally, the results demonstrated that the top five pathways significantly enriched in the high-ICDRLSig risk subgroup were ECM-receptor interaction, focal adhesion, melanoma, pathways in cancer, and regulation of the actin cytoskeleton (Fig. 5I). Our findings suggested that ICDRLSig may be involved in the progression of BC via several tumor-related pathways.

### Mutation and immunity analyses of the ICDRLSig

**Figure 6:**
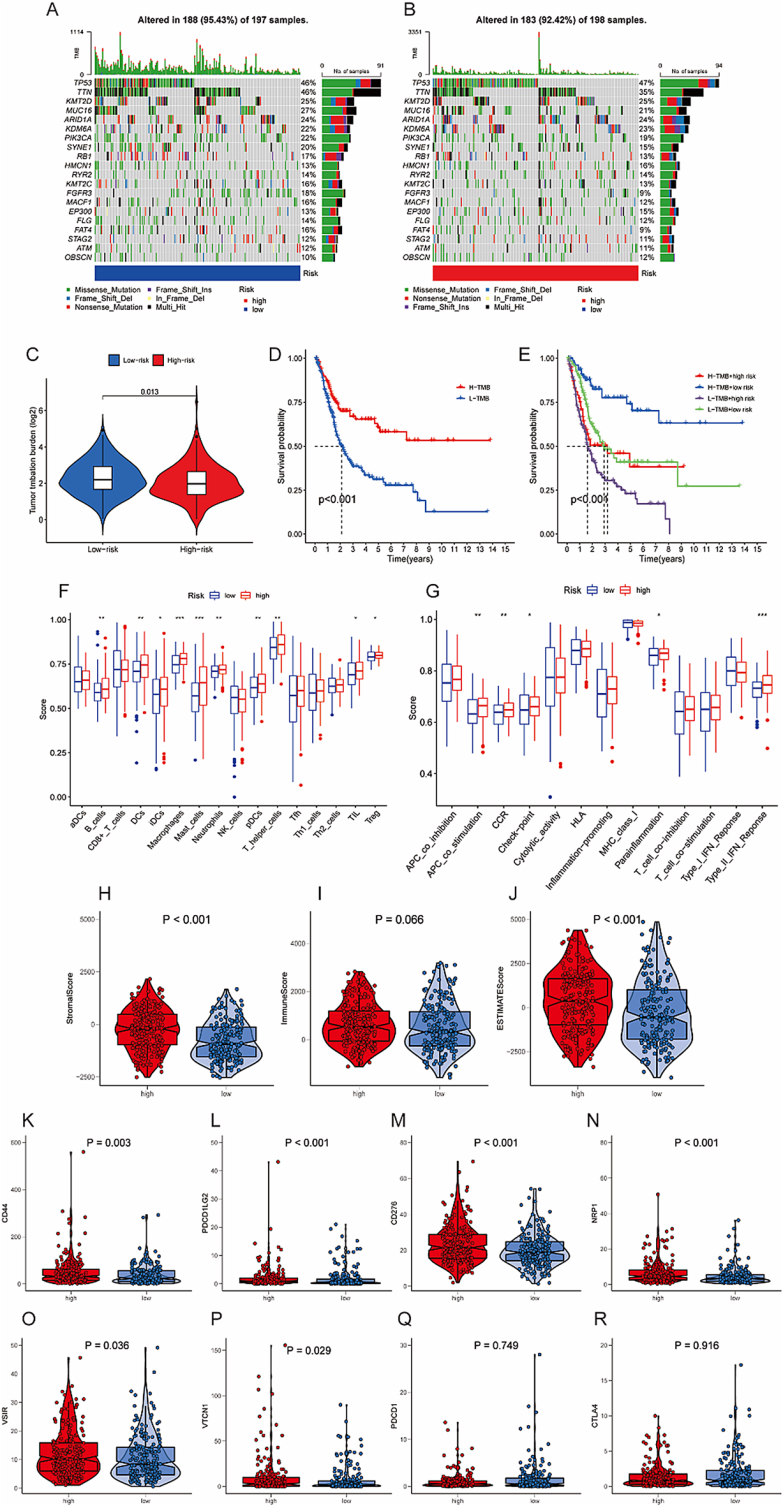
Somatic mutation and immune landscape of immunogenic cell death-related lncRNA signature (ICDRLSig). (**A**, **B**) Genes that were mutated in the TCGA cohort that differed between the low-ICDRLSig and high-ICDRLSig subgroups are shown. (**C**) The total methylbenzofuran (TMB) concentration in the low-risk and high-risk groups. (**D**) A contrast of the prognosis for those in the high TMB grouping with those in the low TMB subgroup. (**E**) A comparison of the prognosis of TMB when risk ratings are taken into account. (**F**–**G**) A subpopulation-specific generalized estimating equation (ssGSEA) for the connection between immune cell subpopulations and related functions (**H**–**R**) The difference in immunity suppressor gene expression between the high-risk group and the low-risk group.

We studied the data on simple nucleotide variation that was provided by the TCGA so that we could investigate the differences in the mutational landscapes of the two groups. The top 5 genes with the highest mutation frequencies in the low-risk group were TP53 (46%), TTN (46%), MUC16 (27%), KMT2D (25%, and ARID1A (24%) (Fig. 6A), whereas the top 5 genes with the highest mutation frequencies in the high-risk group were TP53 (47%), TTN (35%), KMT2D (25%), ARID1A (24%, and KDM6A (23%) (Fig. 6B). Additionally, low-ICDRLSig risk patients had higher TMB relative to high-ICDRLSig risk patients (Fig. 6C). It is worth noting that TMB is an emerging biomarker related to the immunotherapy response, patients with high TMB (H-TMB) generally respond better to immunotherapy than patients with low TMB (L-TMB). In addition, we found that patients with H-TMB had a more favorable survival outcome than patients with L-TMB (Fig. 6D). Moreover, the high-risk & L-TMB group showed worse prognosis compared with the low-risk & H-TMB group (Fig. 6E). According to the findings of our study, people with a reduced ICDRLSig risk could perhaps respond better to immunotherapy. In order to investigate the relationship between the ICDRLSig risk score and the immune landscape of the BC microenvironment, we used SSGSEA to quantify the enrichment scores of a number of different immune cell subpopulations, as well as functions or pathways related to those subpopulations that were found in TCGA. Because of this, we were able to gain a deeper comprehension of the characteristics of this connection. It is worth noting that the subgroup with a high ICDRLSig risk had higher immune cell scores than the subgroup with a low ICDRLSig risk because this finding is interesting. These increased immune cell scores were observed in B cells, DCs, iDCs, macrophages, mast cells, neutrophils, pDCs, T helper cells, TIL, and Treg cells respectively (Fig. 6F). In addition, we discovered that the scores of five immune-related pathways were statistically distinct between the two distinct groups of individuals who were at risk. In the high-ICDRLSig risk grouping, the score of APC co-stimulation, CCR, check-point, parainflammation, and type II IFN response was higher (Fig. 6G). TME score was used to measure differences in the extent of stromal and immune cell infiltration between low- and high-ICDRLSig risk subgroups in order to further investigate the relationship between the ICDRLSig risk score and tumor immune microenvironment in patients with BC. This was done in order to further investigate the relationship between these two factors. Despite the fact that the difference in immune scores between the low- and high-ICDRLSig risk subgroups was not statistically significant, the high-ICDRLSig risk subgroup displayed higher stromal scores, immune scores, and ESTIMATE scores in comparison with the low-ICDRLSig risk group, as shown in Fig. 6H–J. Additionally, we discovered statistically significant variations in the levels of expression of multiple immune checkpoint genes between the high-ICDRLSig risk grouping and the low-ICDRLSig risk subgroup (Fig. 6K–R). CD44, PDCD1LG2, CD276, NRP1, VSIR, and VTCN1 all showed significantly higher levels of expression in the high-ICDRLSig risk subgroup, and the difference between the two groups was statistically significant.

### Chemotherapy and immunotherapy efficacy related to the ICDRLSig

**Figure 7:**
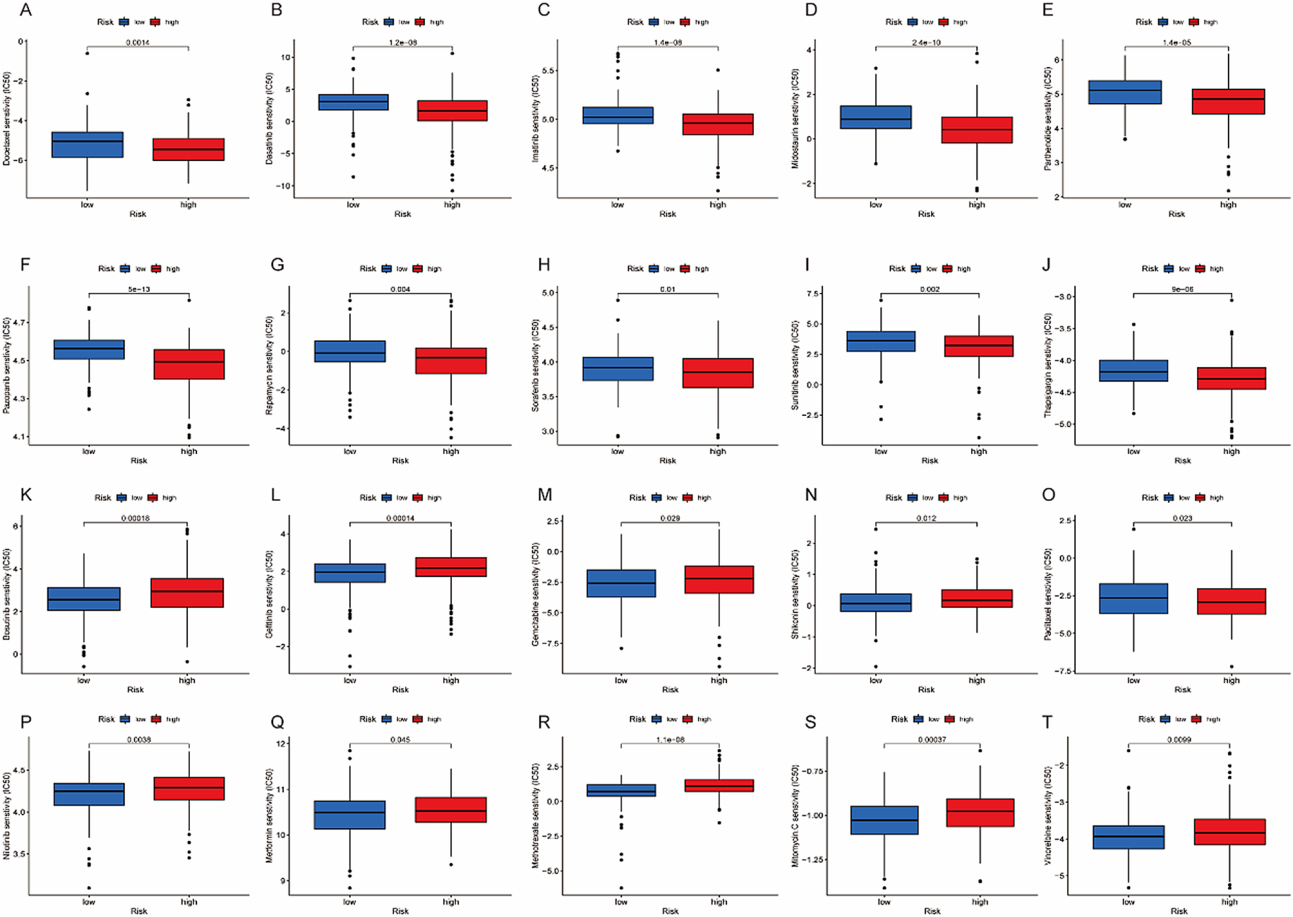
Analysis of chemotherapeutic susceptibility. Difference in the estimated IC50 levels of docetaxel (**A**), dasatinib (**B**), imatinib (**C**), midostaurin (**D**), parthenolide (**E**), pazopanib (**F**), rapamycin (**G**), sorafenib (**H**), sunitinib (**I**), thapsigargin (**J**), bosutinib (**K**), gefitinib (**L**), gemcitabine (**M**), shikonin (**N**), paclitaxel (**O**), nilotinib (**P**), metformin (**Q**), methotrexate (**R**), mitomycin.C (**S**), and vinorelbine (**T**).

**Figure 8:**
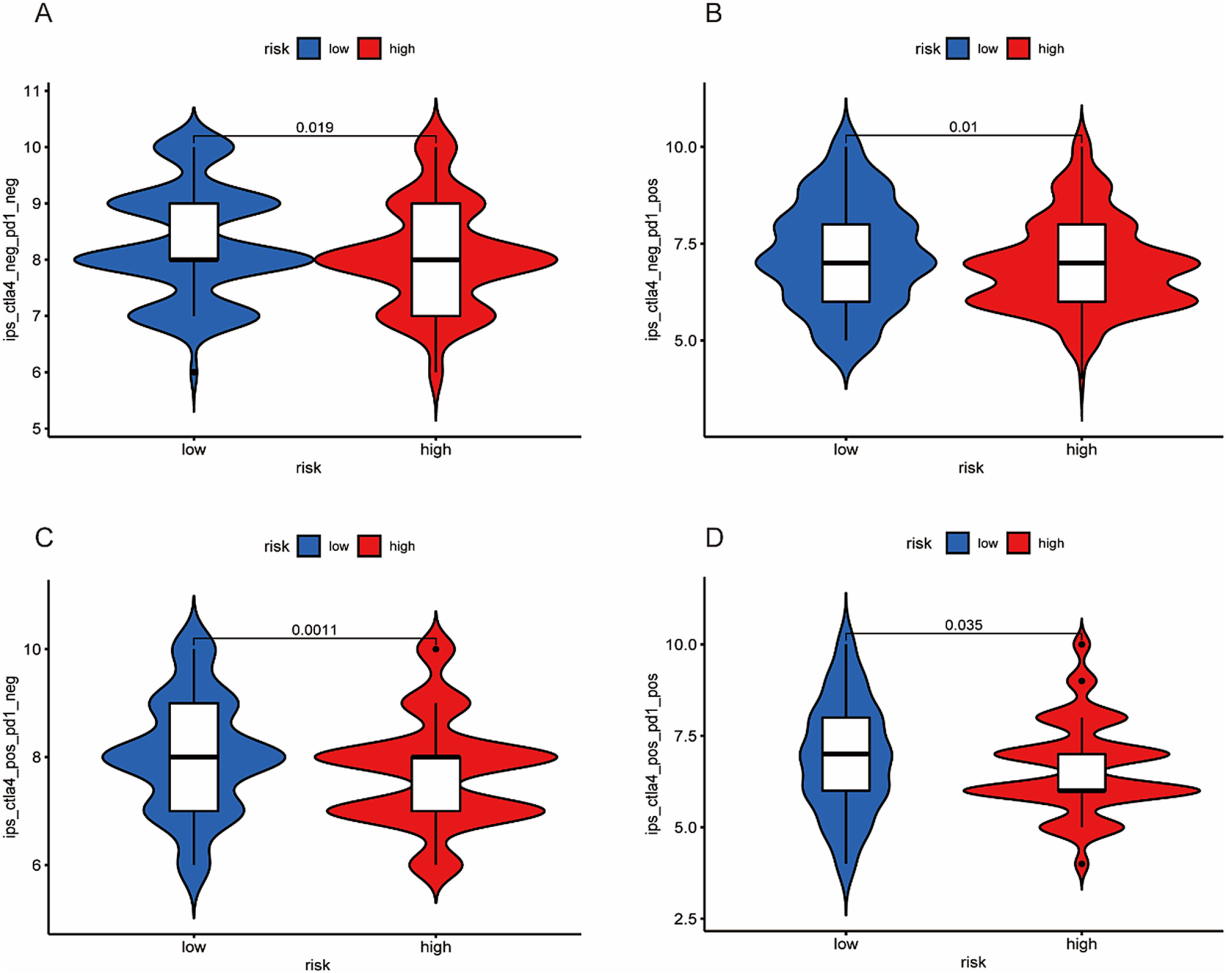
Correlations between the immunogenic cell death-related lncRNA signature (ICDRLSig) and immunotherapy. The ips_ctla4_neg_pd1_neg (**A**), ips_ctla4_neg_pd1_pos (**B**), ips_ctla4_pos_pd1_neg (**C**) and ips_ctla4_pos_pd1_pos (**D**) scores were higher in the low-risk group.

The pRRophetic algorithm was used to calculate IC50 values for 20 prevalent chemotherapeutic agents in the low- and high-ICDRLSig risk subgroups to assess the difference in response to chemotherapy between the two risk subgroups. The IC50 values of docetaxel, dasatinib, imatinib, midostaurin, parthenolide, pazopanib, rapamycin, sorafenib, sunitinib, and thapsigargin in the low-risk subgroup were higher, and these drugs may be better suited for patients in the high-ICDRLSig subgroup (Figs. 7A–J), while low-ICDRLSig risk patients were more sensitive to bosutinib, gefitinib, gemcitabine, shikonin, paclitaxel, nilotinib, metformin, methotrexate, mitomycin.C, and vinorelbine with lower IC50 values (Fig. 7k–T). Additionally, IPS was evaluated in each BC patient to elucidate the immunogenicity of each risk subgroup. As illustrated in Fig. 8A–D, the low-risk group had higher IPS, including ips_ctla4_neg_pd1_neg, ips_ctla4_neg_pd1_pos, ips_ctla4_pos_pd1_neg and ips_ctla4_pos_pd1_pos, indicating that the low-ICDRLSig risk group possessed higher immunogenic properties compared to the high-ICDRLSig risk group.

## Discussion

BC is one of the most common malignancies of the genitourinary system, with the majority originating from the uroepithelium^1^. In routine clinical practice, TMN stage is an important prognostic determinant in BC. However, clinical outcomes of patients with the same stage often differ, suggesting that traditional clinicopathological variables do not adequately predict patient prognosis^7^. Areas under ROC curves were used to evaluate the relative predictive value of risk scores and clinicopathological variables (AUCs). In addition, we investigated the dissimilarities in risk scores between clinical characteristics^32,33^. It has been found that induction of ICD can drive tumour-specific immune responses and influence tumour development^10^. Considering the essential role of immunogenic cell death in cancer, ICDRLSig for predicting patient prognosis is also gaining attention. However, the prognosis of BC patients has not been reported to be predicted by constructing ICDRLSig.

Here, a total of 13 ICDRLs (AC008050.1, AC018926.2, AC125494.1, AL133415.1, AC023825.2, BX005019.1, AC011477.3, LINC00968, AC022150.2, AC022467.1, AC012363.2, AC009299.2, and AC084064.1) was identified to construct a predictive signature. this is the first study to identify and comprehensively investigate the prognostic ICDRLSig in BC. The signature based on 13 ICDRLs provided a useful tool to integrate with traditional clinicopathological factors and guide prognostic prediction and treatment decisions. Additionally, we provide an ICDRLSig-related nomogram to enhance prediction of BC prognosis.

So far, of these 13 ICDRLs, only the biological function of LINC00968 in human cancer pathology has been demonstrated in previous report, such as suppressed tumorigenesis and metastasis of lung adenocarcinoma by silencing miR-9-5p as a molecular sponge^34^. To illustrate the potential molecular mechanisms and biological functions of the established signature, we performed functional enrichment analyses and revealed that the high-ICDRLSig risk group showed significant activation of cancer-associated signaling pathways, such as ECM-receptor interaction, focal adhesion, proteoglycans in cancer, and pathways in cancer.

The immunotherapy known as immune checkpoint blockade (ICB), which is used rather regularly, has shown encouraging clinical outcomes in treating several forms of cancer, while having poor effectiveness overall^35^. In recent years, evidence has accumulated suggesting that lactate plays important functions in the immunological response^36^. High TMB is associated with better treatment outcomes with ICB^37^. In this report, patients in the low-ICDRLSig risk group had higher TMB, and survival outcome revealed that patients with high TMB survived better, indicating a better benefit of immunotherapy in low-ICDRLSig risk patients. The immune microenvironment was able to affect the initiation and progression of tumors^38^. We found that the immunosuppressive nature of the tumor immune microenvironment may contribute to the unfavorable prognosis of patients with high-ICDRLSig risk, as we observed higher levels of immune cell infiltration, including immunosuppressive cells (Tregs), in high-ICDRLSig risk samples. We also found higher expression levels of immune checkpoints, such as CD44, PDCD1LG2, CD276, NRP1, VSIR, and VTCN1.

Chemotherapy has become an effective therapeutic strategy for BC^39^. Our research found ten common sensitive chemotherapeutic drugs in the low-ICDRLSig risk and high-ICDRLSig risk subgroups, respectively. The low-ICDRLSig risk group was more sensitive to bosutinib, gefitinib, gemcitabine, shikonin, paclitaxel, nilotinib, metformin, methotrexate, mitomycin.C, and vinorelbine, while docetaxel, dasatinib, imatinib, midostaurin, parthenolide, pazopanib, rapamycin, sorafenib, sunitinib, and thapsigargin are more suitable for high-ICDRLSig risk patients. In addition, IPS analysis was applied to analyze and compare the immunogenicity of the two risk subgroups. The low-ICDRLSig risk group had higher scores for ips_ctla4_neg_pd1_pos, ips_ctla4_pos_pd1_neg, and ips_ctla4_pos_pd1_pos, suggesting that patients in the low-ICDRLSig risk subgroup were more immunogenic and responded better to immunotherapy.

## Conclusion

Overall, an immunogenic cell death-related 13-lncRNA signature was successfully created in this study to predict the prognosis of patients with BC. The study still has limitations and weaknesses. Firstly, since other databases do not contain lncRNA expression profiles and OS data, external validation is not possible. Secondly, these analysis results currently lack effective experimental verification, and their biological roles need to be further clarified. Finally, the interaction between immunogenic cell death-related lncRNAs and the BC immune microenvironment needs to be better understood.

### Supplementary Information

Supplementary Tables.

## Data Availability

The datasets generated during and/or analyzed during the current study are available from the corresponding author upon reasonable request.

## References

[1] DeGeorge KC, Holt HR, Hodges SC (2017). Bladder cancer: Diagnosis and treatment. Am. Fam. Phys..

[2] Patel VG, Oh WK, Galsky MD (2020). Treatment of muscle-invasive and advanced bladder cancer in 2020. CA: Cancer J. Clin..

[3] Han J, Gu X, Li Y, Wu Q (2020). Mechanisms of BCG in the treatment of bladder cancer-current understanding and the prospect. Biomed.Pharmacother. Biomed. Pharmacother..

[4] Sánchez Vázquez A, Pereira Arias JG, Gamarra Quintanilla M, Urdaneta Salegui F, Mora Christian J, Ibarluzea González G, Astobieta Odriozola A (2018). BCG therapy in NMIBC: How much and for how long?. Archivos Espanoles de Urologia.

[5] Shore ND, Palou Redorta J, Robert G, Hutson TE, Cesari R, Hariharan S, Rodríguez Faba Ó, Briganti A, Steinberg GD (2021). Non-muscle-invasive bladder cancer: An overview of potential new treatment options. Urol. Oncol..

[6] Racioppi M (2021). Advances in management of bladder cancer. J. Clin. Med..

[7] Dobruch J, Oszczudłowski M (2021). Bladder cancer: Current challenges and future directions. Medicina.

[8] Ulamec M, Murgić J, Novosel L, Tomić M, Terlević R, Tomašković I, Jazvić M, Froebe A, Krušlin B (2021). New insights into the diagnosis, molecular taxonomy, and treatment of bladder cancer. Acta Med. Acad..

[9] Taskovska M, Kreft ME, Smrkolj T (2020). Current and innovative approaches in the treatment of non-muscle invasive bladder cancer: The role of transurethral resection of bladder tumor and organoids. Radiol. Oncol..

[10] Ahmed A, Tait SWG (2020). Targeting immunogenic cell death in cancer. Mol. Oncol..

[11] Galluzzi L, Vitale I, Warren S, Adjemian S, Agostinis P, Martinez AB, Chan TA, Coukos G, Demaria S, Deutsch E, Draganov D, Edelson RL, Formenti SC, Fucikova J, Gabriele L, Gaipl US, Gameiro SR, Garg AD, Golden E, Han J, Harrington KJ, Hemminki A, Hodge JW, Hossain DMS, Illidge T, Karin M, Kaufman HL, Kepp O, Kroemer G, Lasarte JJ, Loi S, Lotze MT, Manic G, Merghoub T, Melcher AA, Mossman KL, Prosper F, Rekdal Ø, Rescigno M, Riganti C, Sistigu A, Smyth MJ, Spisek R, Stagg J, Strauss BE, Tang D, Tatsuno K, van Gool SW, Vandenabeele P, Yamazaki T, Zamarin D, Zitvogel L, Cesano A, Marincola FM (2020). Consensus guidelines for the definition, detection and interpretation of immunogenic cell death. J. Immunother. Cancer.

[12] Zhou J, Wang G, Chen Y, Wang H, Hua Y, Cai Z (2019). Immunogenic cell death in cancer therapy: Present and emerging inducers. J. Cell Mol. Med..

[13] Fucikova J, Kepp O, Kasikova L, Petroni G, Yamazaki T, Liu P, Zhao L, Spisek R, Kroemer G, Galluzzi L (2020). Detection of immunogenic cell death and its relevance for cancer therapy. Cell Death Dis..

[14] D'Eliseo D, Manzi L, Velotti F (2013). Capsaicin as an inducer of damage-associated molecular patterns (DAMPs) of immunogenic cell death (ICD) in human bladder cancer cells. Cell Stress Chaperones.

[15] Xu L, Su B, Mo L, Zhao C, Zhao Z, Li H, Hu Z, Li J (2022). Norcantharidin induces immunogenic cell death of bladder cancer cells through promoting autophagy in acidic culture. Int. J. Mol. Sci..

[16] Bridges MC, Daulagala AC, Kourtidis A (2021). LNCcation: lncRNA localization and function. J. Cell Biol..

[17] Ferrè F, Colantoni A, Helmer-Citterich M (2016). Revealing protein-lncRNA interaction. Brief. Bioinform..

[18] Bhan A, Soleimani M, Mandal SS (2017). Long Noncoding RNA and cancer: A new paradigm. Cancer Res..

[19] Zhang Y, Chen X, Lin J, Jin X (2021). Biological functions and clinical significance of long noncoding RNAs in bladder cancer. Cell Death Discov..

[20] Zhang X, Ren L, Yan X, Shan Y, Liu L, Zhou J, Kuang Q, Li M, Long H, Lai W (2020). Identification of immune-related lncRNAs in periodontitis reveals regulation network of gene-lncRNA-pathway-immunocyte. Int. Immunopharmacol..

[21] Luo W, Wang J, Xu W, Ma C, Wan F, Huang Y, Yao M, Zhang H, Qu Y, Ye D, Zhu Y (2021). LncRNA RP11-89 facilitates tumorigenesis and ferroptosis resistance through PROM2-activated iron export by sponging miR-129-5p in bladder cancer. Cell Death Dis..

[22] Quan J, Pan X, Zhao L, Li Z, Dai K, Yan F, Liu S, Ma H, Lai Y (2018). LncRNA as a diagnostic and prognostic biomarker in bladder cancer: A systematic review and meta-analysis. OncoTargets Ther..

[23] Garg AD, De Ruysscher D, Agostinis P (2016). Immunological metagene signatures derived from immunogenic cancer cell death associate with improved survival of patients with lung, breast or ovarian malignancies: A large-scale meta-analysis. Oncoimmunology.

[24] Kim WJ, Kim EJ, Kim SK, Kim YJ, Ha YS, Jeong P, Kim MJ, Yun SJ, Lee KM, Moon SK, Lee SC, Cha EJ, Bae SC (2010). Predictive value of progression-related gene classifier in primary non-muscle invasive bladder cancer. Mol. Cancer.

[25] Yu G, Wang LG, Han Y, He QY (2012). clusterProfiler: an R package for comparing biological themes among gene clusters. Omics J. Integr. Biol..

[26] Hänzelmann S, Castelo R, Guinney J (2013). GSVA: Gene set variation analysis for microarray and RNA-seq data. BMC Bioinform..

[27] Yoshihara K, Shahmoradgoli M, Martínez E, Vegesna R, Kim H, Torres-Garcia W, Treviño V, Shen H, Laird PW, Levine DA, Carter SL, Getz G, Stemke-Hale K, Mills GB, Verhaak RG (2013). Inferring tumour purity and stromal and immune cell admixture from expression data. Nat. Commun..

[28] Mayakonda A, Lin DC, Assenov Y, Plass C, Koeffler HP (2018). Maftools: Efficient and comprehensive analysis of somatic variants in cancer. Genome Res..

[29] Geeleher P, Cox N, Huang RS (2014). pRRophetic: An R package for prediction of clinical chemotherapeutic response from tumor gene expression levels. PloS ONE.

[30] Charoentong P, Finotello F, Angelova M, Mayer C, Efremova M, Rieder D, Hackl H, Trajanoski Z (2017). Pan-cancer immunogenomic analyses reveal genotype-immunophenotype relationships and predictors of response to checkpoint blockade. Cell Rep..

[31] Sun X, Xin S, Jin L, Zhang Y, Ye L (2022). Neurexophilin 4 is a prognostic biomarker correlated with immune infiltration in bladder cancer. Bioengineered.

[32] Li B, Cui Y, Diehn M, Li R (2017). Development and validation of an individualized immune prognostic signature in early-stage nonsquamous non-small cell lung cancer. JAMA Oncol..

[33] Danaher P, Warren S, Lu R, Samayoa J, Sullivan A, Pekker I, Wallden B, Marincola FM, Cesano A (2018). Pan-cancer adaptive immune resistance as defined by the Tumor Inflammation Signature (TIS): Results from The Cancer Genome Atlas (TCGA). J. Immunother. Cancer.

[34] Tang H, Han X, Feng Y, Hao Y (2020). linc00968 inhibits the tumorigenesis and metastasis of lung adenocarcinoma via serving as a ceRNA against miR-9-5p and increasing CPEB3. Aging.

[35] Morad G, Helmink BA, Sharma P, Wargo JA (2021). Hallmarks of response, resistance, and toxicity to immune checkpoint blockade. Cell.

[36] Ippolito L, Morandi A, Giannoni E, Chiarugi P (2019). Lactate: A metabolic driver in the tumour landscape. Trends Biochem. Sci..

[37] Boumber Y (2018). Tumor mutational burden (TMB) as a biomarker of response to immunotherapy in small cell lung cancer. J. Thorac. Dis..

[38] Hinshaw DC, Shevde LA (2019). The tumor microenvironment innately modulates cancer progression. Cancer Res..

[39] Ismaili N, Amzerin M, Flechon A (2011). Chemotherapy in advanced bladder cancer: Current status and future. J. Hematol. Oncol..

