# Supplementary material for: An immunogenic cell death-related lncRNA signature correlates with prognosis and tumor immune microenvironment in bladder cancer
Source: Sci Rep. 2024 Jun 7;14:13106. doi: 10.1038/s41598-024-63852-9 (PMC11161581; doi:10.1038/s41598-024-63852-9)
Supplement: Supplementary file 1 — Supplementary Tables. [file 41598_2024_63852_MOESM1_ESM.docx]

Table S1: The names of all ICD-related genes.

ENTPD1

NT5E

CALR

HMGB1

HSP90AA1

ATG5

BAX

CASP8

PDIA3

EIF2AK3

PIK3CA

CXCR3

IFNA1

IFNB1

IL10

IL6

TNF

CASP1

IL1R1

IL1B

NLRP3

P2RX7

LY96

MYD88

TLR4

FOXP3

IFNG

IFNGR1

IL17A

IL17RA

PRF1

CD4

CD8A

CD8B

Table S2: 19 prognosis-associated lncRNAs in the train set through univariable Cox analysis.

| Gene | HR | P-value | Lower | Upper |
| --- | --- | --- | --- | --- |
| AC008050.1 | 4.2463 | 0.0004 | 1.9218 | 9.3823 |
| AC018926.2 | 0.5853 | 0.0013 | 0.4223 | 0.8113 |
| AC125494.1 | 0.0201 | 0.0084 | 0.0011 | 0.3677 |
| AL133415.1 | 2.6270 | 0.0060 | 1.3186 | 5.2333 |
| AC023825.2 | 0.1691 | 0.0095 | 0.0441 | 0.6476 |
| BX005019.1 | 35.3050 | 0.0078 | 2.5615 | 486.6123 |
| LINC02207 | 3.2232 | 0.0003 | 1.7074 | 6.0850 |
| AC011477.3 | 0.8793 | 0.0060 | 0.8021 | 0.9638 |
| LINC01936 | 4.8605 | 0.0038 | 1.6680 | 14.1630 |
| AC021546.1 | 31.9380 | 0.0072 | 2.5554 | 399.1622 |
| LINC00968 | 3.2351 | 0.0002 | 1.7488 | 5.9846 |
| AL583785.1 | 1.0707 | 0.0030 | 1.0235 | 1.1200 |
| AC022150.2 | 0.8603 | 0.0042 | 0.7761 | 0.9537 |
| AC008543.3 | 0.3429 | 0.0045 | 0.1640 | 0.7170 |
| AL160153.1 | 1.4212 | 0.0002 | 1.1780 | 1.7146 |
| AC022467.1 | 3.2467 | 0.0014 | 1.5769 | 6.6845 |
| AC012363.2 | 1.9211 | 0.0090 | 1.1774 | 3.1345 |
| AC009299.2 | 1.6424 | 0.0025 | 1.1901 | 2.2665 |
| AC084064.1 | 8.5610 | 0.0027 | 2.1056 | 34.8069 |
